# Acute Liver Failure in a Patient Treated With Metamizole

**DOI:** 10.3389/fphar.2019.00996

**Published:** 2019-09-11

**Authors:** Philipp Krisai, Deborah Rudin, David Grünig, Kathrin Scherer, Werner Pichler, Luigi Terracciano, Stephan Krähenbühl

**Affiliations:** ^1^Cardiovascular Research Institute Basel, University Hospital Basel, Basel, Switzerland; ^2^Division of Clinical Pharmacology & Toxicology, University Hospital Basel, Basel, Switzerland; ^3^Allergy Unit, Department of Dermatology, University Hospital Basel, Basel, Switzerland; ^4^ADR-AC GmbH, Berne, Switzerland; ^5^Institute of Pathology, University Hospital Basel, Basel, Switzerland

**Keywords:** metamizole, *N*-methyl-4-aminoantipyrine (MAA), 4-aminoantipyrine (AA), liver failure, lymphocyte transformation test (LTT)

## Abstract

We report on a patient who developed acute liver failure while being treated with metamizole. After liver transplantation, the patient recovered rapidly. Liver biopsy showed massive necrosis and lobular infiltration of lymphocytes. A lymphocyte transformation test performed 20 months after transplantation was positive for metamizole. *In vitro* investigations with *N*-methyl-4-aminoantipyrine (MAA) and 4-aminoantipyrine (AA), the two active metabolites of metamizole, did not reveal relevant toxicity in HepG2 and HepaRG cells. The demonstration of activated lymphocytes by the lymphocyte transformation test and the absence of relevant cytotoxicity by MAA and AA in hepatocyte cell lines suggest an immunological mechanism of metamizole-associated hepatotoxicity.

## Introduction

**Figure 1:**
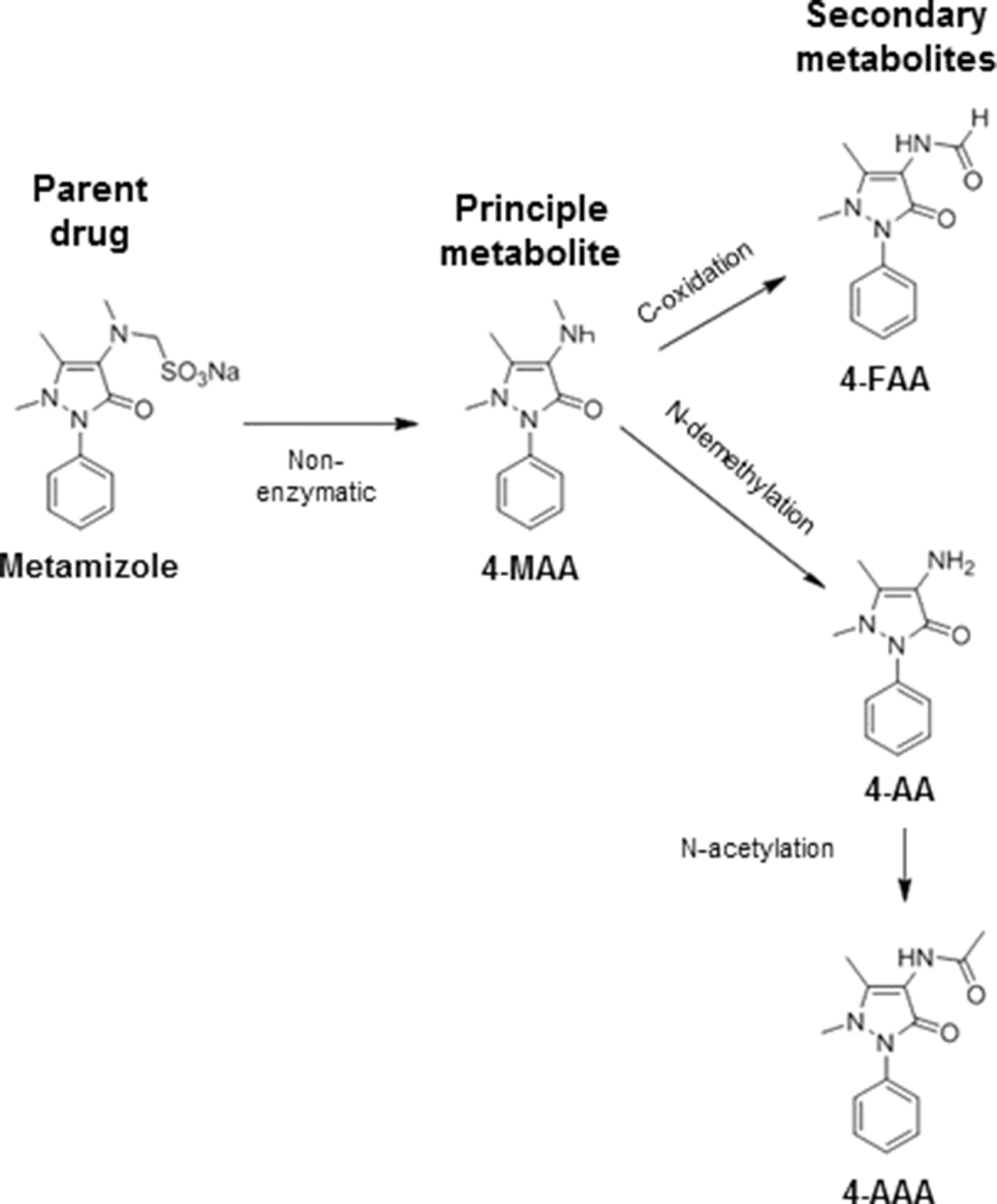
Metabolism of metamizole. Metamizole is converted non-enzymatically to *N*-methyl-4-aminoantipyrine (MAA), which is the most important active metabolite. MMA can be demethylated to 4-aminoantipyrine (AA) or oxidized to *N*-formyl-4-aminoantipyrine (FAA). AA can also be acetylated to *N*-acetyl-4-aminoantiyprine (AAA).

Metamizole (dipyrone) is a non-opioid analgesic and antipyretic drug with an analgesic efficacy stronger than paracetamol and at least as strong as ibuprofen (Gomez-Jimenez et al., 1980; Planas et al., 1998). As shown in **Figure 1**, metamizole is a prodrug that is metabolized non-enzymatically almost quantitatively to *N*-methyl-4-aminoantipyrine (MAA), which has an oral bioavailability close to 100%. MAA is further metabolized by *N*-demethylation to 4-aminoantipyrine (AA) and by formylation to *N*-formyl-4-aminoantipyrine (FAA). In addition, AA can be metabolized by *N*-acetylation to *N*-acetyl-4-aminoantipyrine (AAA) (Zylber-Katz et al., 1992; Bachmann et al., 2018). Both MAA and AA are active (Pierre et al., 2007), but the exposure to AA is approximately five times lower than for MAA (Zylber-Katz et al., 1992), indicating that the analgesic activity of metamizole is mainly due to MAA. Maximal serum concentrations of MAA reached after oral ingestion of a typical dose of 1 g are in the range of 10 to 20 µg/ml (Zylber-Katz et al., 1992), corresponding to 50 to 100 µM. The volume of distribution of MAA is in the range of 1 L/kg, suggesting that the tissue concentrations are close to the serum concentrations.

Metamizole is usually well tolerated but can rarely lead to neutropenia and agranulocytosis, which may be life threatening (Hedenmalm and Spigset, 2002; Blaser et al., 2015; Huber et al., 2015). The risk for agranulocytosis is the reason why metamizole was withdrawn from the market in many countries. Additional adverse reactions include allergic reactions manifesting as skin eruptions (Borja et al., 2003; Blanca-Lopez et al., 2016) or anaphylaxis with arterial hypotension (Garcia-Martin et al., 2015; Andrade et al., 2016). Liver injury has been reported (Federmann et al., 1988; Herdeg et al., 2002) but appears to be extremely rare. We report on a patient treated with metamizole who developed fulminant liver injury. The investigation of the mechanism of liver toxicity suggested an immunological mechanism.

## Case Presentation

We report the case of a 54-year-old Caucasian female presenting with liver failure while being treated with metamizole. Metamizole (4 g/day) was started after mastectomy and hysterectomy 2 months before presentation and was continued until the actual presentation. Mastectomy and hysterectomy were performed due to high-risk genetics. Other medications were stopped during hospital stay (acetylsalicylic acid) or 2 weeks after surgical intervention (clindamycin, dalteparin, and esomeprazole), and no other drugs or food supplements were taken in the meantime.

**Figure 2:**
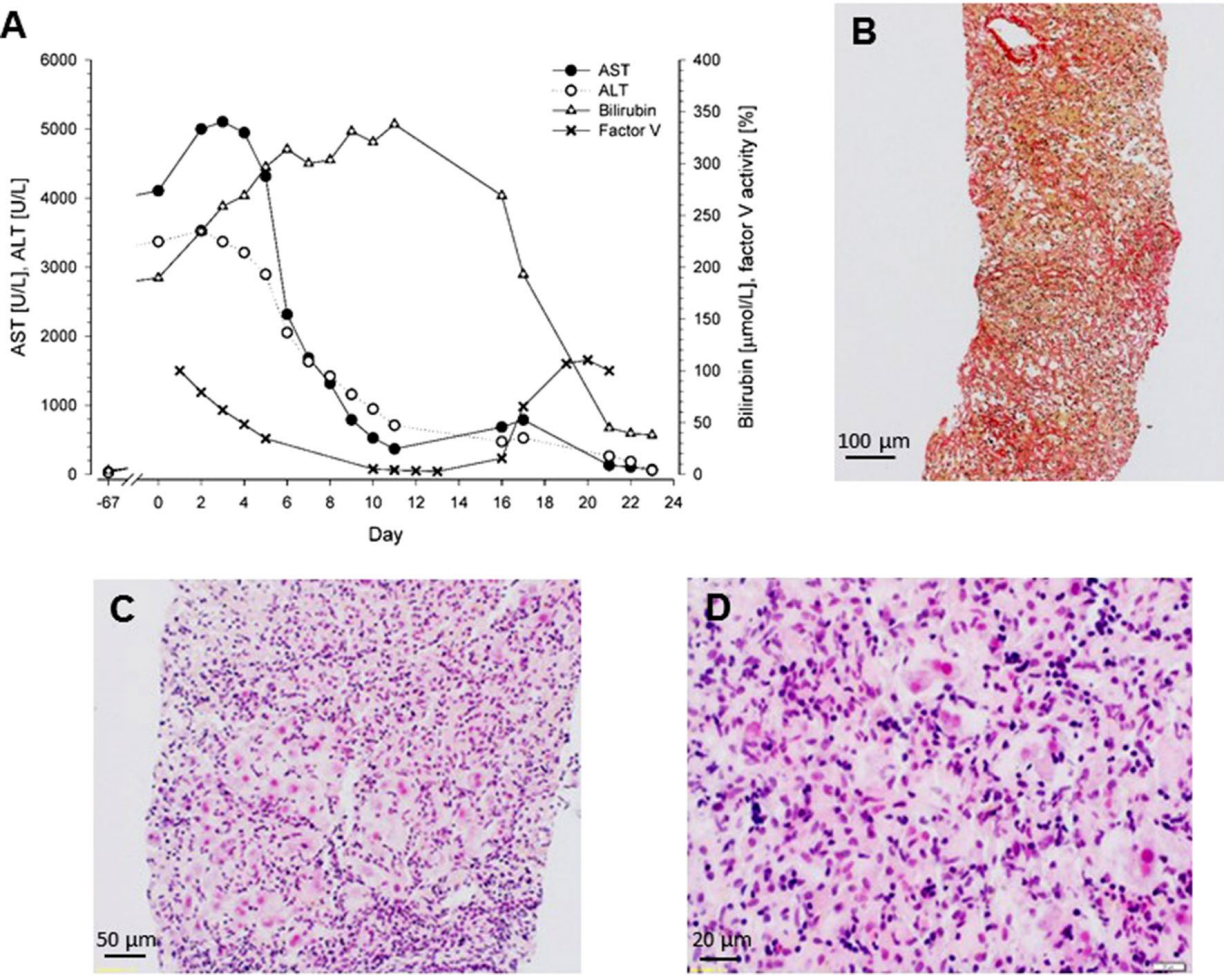
**(A)** Biochemical parameters of a patient with acute liver failure caused by metamizole. Liver transplantation was performed at day 16. **(B)** Liver biopsy obtained at day 4, Sirius Red staining. Collapse and condensation of the preexisting reticulin framework. **(C)** Liver biopsy obtained at day 4, hematoxylin and eosin staining. Dropout of extensive areas of liver cells with few surviving hepatocytes mainly in the periportal area and severe lobular inflammation. **(D)** Liver biopsy obtained at day 4, hematoxylin and eosin staining. The lobular infiltrate consists of mainly lymphocytes and histiocytes; eosinophils are not present.

The patient reported malaise over the last 3 weeks and dark urine and jaundice that had developed gradually over the week before presentation. The physical examination was normal, and there was no exanthema. Viral (hepatitis A, B, C, and E) and autoimmune hepatitis were excluded *via* serology or polymerase chain reaction, as appropriate. Furthermore, cytomegalovirus (CMV), Epstein–Barr virus (EBV), and human immunodeficiency virus (HIV) as well as hemochromatosis, Wilson disease, and alpha-1 antitrypsin deficiency were excluded. The patient had no history of alcohol or drug abuse, toxicological screening was unremarkable, and a normal Doppler ultrasound excluded hemodynamic liver injury. Blood analysis at admission revealed massive elevation of transaminases [aspartate transaminase (AST) 4,104 U/L and alanine aminotransferase (ALT) 3,375 U/L], bilirubin (195 µmol/L), cholestasis parameters [gamma-glutamyl transferase (GGT) 1,061 U/L and alkaline phosphatase 586 U/L), and impaired coagulation [international normalized ratio (INR) 1.4] (**Figure 2A**). There was no eosinophilia. A liver biopsy was obtained showing acute hepatitis with diffuse panlobular, massive necrosis, and severe collapse of reticulin fibers. The histological picture was characterized by the collapse and condensation of the preexisting reticulin framework (**Figure 2B**) and by the dropout of extensive areas of liver cells with only few, mostly periportal, surviving hepatocytes (**Figure 2C**). There was a severe lobular inflammation with lymphocytes and histiocytes, but no eosinophils (**Figure 2D**).

As shown in **Figure 2A**, transaminase levels peaked at day 5 and then decreased, probably reflecting complete loss of functional hepatocytes, and the serum bilirubin concentration increased. The factor V activity decreased to less than 10% of normal, reflecting the strongly impaired synthetic capacity of the liver. The patient was listed for super urgent liver transplantation and received the donor organ on day 16. After transplantation, all blood values gradually returned to the normal range (**Figure 2A**), and 3 months after transplantation, all parameters of liver function had normalized.

Twenty months after liver transplantation, a lymphocyte transformation test (LTT) was performed, which included metamizole, clindamycin, and esomeprazole. At this time point, the patient was treated with tacrolimus (4 mg q12h) and amlodipine (10 mg q24h). The test was positive for metamizole and negative for clindamycin and esomeprazole, supporting the causality assessment that metamizole was the culprit and suggesting an immunological mechanism.

The causality assessment using the Roussel Uclaf Causality Assessment Method (RUCAM) score resulted in 11 points, which means “highly probable” (Danan and Benichou, 1993).

The patient gave written consent for the publication of the data contained in this report.

## Methods

The LTT was performed as described previously by Pichler and Tilch (2004). Lymphocytes were isolated from whole blood and kept at a density of 2 × 10^6^/ml in RPMI-1640 medium supplemented with HEPES buffer and 20% AB serum. Lymphocyte suspension of 100 µl (2 × 10^5^ cells) was placed in flat-bottom wells of a microtiter plate, the drugs to be investigated or the positive control (tetanus toxoid 1 µg/ml final concentration) was added, and the cell suspensions were kept in a 5% CO_2_ ventilated incubator for 5 days at 37°C. At day 5, ^3^H-thymidine was added overnight, and cells were harvested the next morning for β-counting. Unspecific stimulation of T-cell proliferation with pokeweed mitogen (1 µg/ml final concentration) was used as a quality control for T-cell reactivity. Incubations were performed in triplicate, and the standard deviation of the determinations was less than 30% of the mean. Results are given as a stimulation index (SI), which corresponds to the ratio of dpm in the presence of a specific drug divided by the dpm of incubations without this drug.

In addition, we performed toxicological *in vitro* investigations in order to test the hypothesis that metamizole could cause liver injury by a direct toxic mechanism. For that, we used HepG2 cells and HepaRG cells, two well-established human hepatoma cell lines (Berger et al., 2016). Cell culture and the determination of the cellular adenosine triphosphate (ATP) content (a marker of mitochondrial function and cell viability) and release of adenylate kinase (a marker of plasma membrane intactness) were carried out using commercial kits as described previously (Rudin et al., 2019b). Cytochrome P450 enzyme (CYP) induction was achieved by pretreatment of HepaRG cells with 20 µM of rifampicin for 2 days (Berger et al., 2016). Rifampicin induced the CYP3A4 content by a factor of 29, which is comparable to our previous study (Berger et al., 2016). The results of the toxicological experiments are displayed as mean ± standard deviation of at least three independent experiments. Multiple means were analyzed by analysis of variance (ANOVA). In case of significant differences between groups, significance against control incubations was assessed using unpaired *t*-tests with Bonferroni correction. *P* < 0.05 was considered as a significant difference.

Statistical analyses were performed using GraphPad Prism 6 (GraphPad Software, La Jolla, CA, USA).

## Results

The LTT had to be performed while the patient was under immunosuppression with oral tacrolimus (4 mg every 12 h), which impairs the proliferation of T cells. Metamizole was associated with a relevant increase in the SI of the LTT at the highest concentration (SI 1.3 at 1.0 µg/ml, 1.2 at 10.0 µg/ml, and 2.5 at 50 µg/ml), whereas other drugs tested showed no significant stimulation at the highest concentration (SI for clindamycin 1.5 and for esomeprazole 1.2). The SI was 2.5 for tetanus toxoid and 112 for pokeweed mitogen.

**Figure 3:**
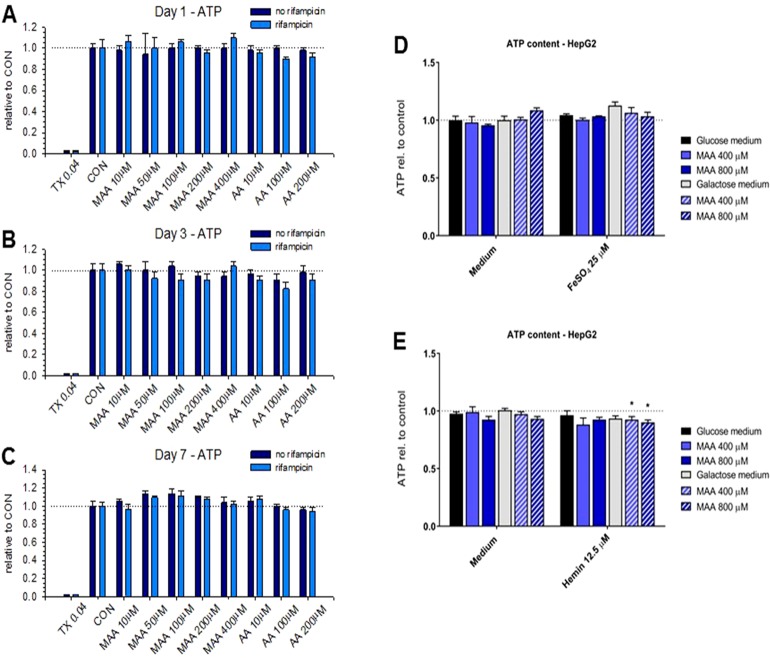
**(A** to **C)** Cellular adenosine triphosphate (ATP) content of HepaRG cells with or without pretreatment with rifampicin (20 μM for 2 days) for cytochrome P450 enzyme (CYP) induction. Neither treatment with *N*-methyl-4-aminoantipyrine (MAA, 10–400 μM) nor with 4-aminantipyrine (4-AA, 10–200 μM) for 1 to 7 days reduced the cellular ATP content significantly irrespective of the pretreatment with rifampicin. **(D)** Effect of *N*-methyl-4-aminoantipyrine (MAA, 400–800 μM) on the ATP content of HepG2 cells grown under glucose or galactose conditions in the absence or presence of FeSO_4_ (25 μM). **(E)** Effect of *N*-methyl-4-aminoantipyrine (MAA, 400–800 μM) on the ATP content of HepG2 cells grown under glucose or galactose conditions in the absence or presence of hemin (12.5 μM). The data in panels **(D** and **E)** show no relevant increase in the toxicity of MAA or AA in the presence of iron or under conditions favoring mitochondrial ATP production (incubations with galactose). Abbreviations: TX 0.04, 0.04% Triton X (positive control); CON, control incubations with medium only; MAA, *N*-methyl-4-aminoantipyrine; AA, 4-aminoantipyrine.

**Figure 4:**
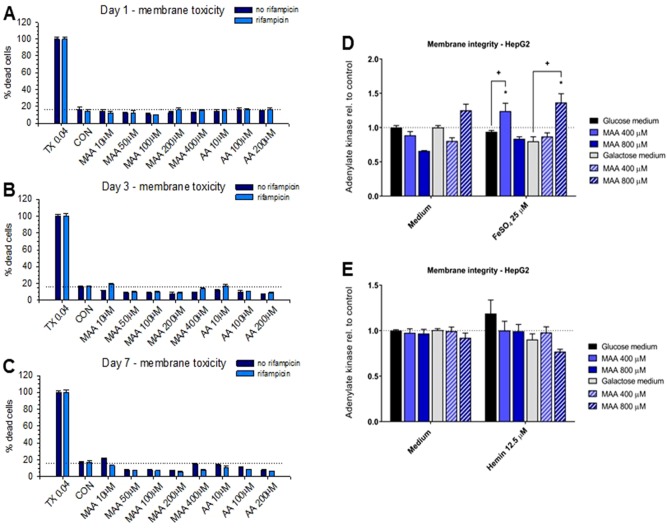
**(A** to **C)** Plasma membrane integrity determined as the release of adenylate kinase of HepaRG cells with or without pretreatment with rifampicin (20 μM for 2 days) for cytochrome P450 enzyme (CYP) induction. Neither treatment with *N*-methyl-4-aminoantipyrine (MAA, 10–400 μM) nor with 4-aminantipyrine (4-AA, 10–200 μM) for 1 to 7 days significantly increased the permeability of the plasma membrane irrespective of pretreatment with rifampicin. **(D)** Effect of *N*-methyl-4-aminoantipyrine (MAA, 400–800 μM) on the plasma membrane permeability of HepG2 cells grown under glucose or galactose conditions in the absence or presence of FeSO_4_ (25 μM). **(E)** Effect of *N*-methyl-4-aminoantipyrine (MAA, 400–800 μM) on the plasma membrane permeability of HepG2 cells grown under glucose or galactose conditions in the absence or presence of hemin (12.5 μM). The data in panels **(D** and **E)** show no relevant increase in the toxicity of MAA or AA in the presence of iron or under conditions favoring mitochondrial ATP production (incubations with galactose). Abbreviations: TX 0.04, 0.04% Triton X (positive control); CON, control incubations with medium only; MAA, *N*-methyl-4-aminoantipyrine; AA, 4-aminoantipyrine.

We have shown recently that MAA, the principal metabolite of metamizole, can exhibit a direct toxic effect on HL60 cells, a granulocyte precursor cell line (Rudin et al., 2019a; Rudin et al., 2019b). Since this could also be the case for hepatocytes, we decided to investigate the toxicity of the metamizole metabolites MAA and AA on hepatocyte cell lines. For that, we exposed HepaRG cells and HepG2 cells to different concentrations of MAA and AA, an active metabolite of MAA (**Figure 1**). In a first experiment, we investigated the cytotoxicity of MAA and AA in HepaRG cells with or without pretreatment with rifampicin as a CYP inducer (Berger et al., 2016) for different time points. As shown in **Figures 3A–C**, we observed no significant effect on the cellular ATP content irrespective of CYP induction, suggesting that MAA, AA, or the possible metabolites of these compounds did not impair mitochondrial function or cell viability. Similarly, neither MAA nor AA increased the permeability of the plasma membrane of HepaRG cells irrespective of CYP induction (**Figure 4A–C**).

Since we and others have shown that MAA can form reactive metabolites in the presence of iron ions (Pierre et al., 2007; Rudin et al., 2019b), we investigated the possibility that MAA is more toxic in the presence of Fe^2+^ or hemin (containing Fe^3+^ complexed in heme). For that, we used HepG2 cells cultured in the presence of glucose or galactose. Galactose forces the cells to rely on mitochondrial (and not glycolytic) ATP generation, which increases the toxicity of toxicants impairing mitochondrial function (Kamalian et al., 2015). As shown in **Figure 3D**, MAA did not deplete the ATP content of HepG2 cells in the presence of Fe^2+^. In the presence of hemin, MAA significantly decreased the cellular ATP content by approximately 10% in the presence of galactose, but not of glucose (**Figure 3E**). In the presence of Fe^2+^, MAA slightly increased the membrane permeability of HepG2 cells under glucose and galactose conditions (**Figure 4D**). In contrast, in the presence of hemin, MAA did not significantly affect membrane permeability of HepG2 cells (**Figure 4E**).

## Discussion

There are currently two reports in the literature that metamizole can be hepatotoxic (Federmann et al., 1988; Herdeg et al., 2002). Similar to our findings, a positive lymphocyte transformation test was found in both patients described so far with metamizole-associated liver injury, suggesting a delayed hypersensitivity reaction with T-cell activation as a mechanism of hepatotoxicity. One of the previously reported patients presented with eosinophilia and exanthema in combination with cholestatic liver injury (Herdeg et al., 2002), a clinical picture compatible with a DRESS (drug rash with eosinophilia and systemic symptom) syndrome. The DRESS syndrome is considered to be a T-cell-mediated, systemic reaction to certain drugs (Pichler et al., 2017). The reactive T cells are cytotoxic and can destroy keratinocytes and other cells, including hepatocytes and cholangiocytes. The patient described in the current report and the second patient with a positive lymphocyte transformation test described previously (Federmann et al., 1988) had no eosinophilia and/or exanthema, despite lymphocyte activation in the presence of metamizole. Obviously, in these two patients, the activated T cells only reacted against hepatocytes and possibly cholangiocytes, but not against keratinocytes.

In the patient described in the current report, the stimulation of lymphocyte proliferation by metamizole in the LTT was borderline when considering the absolute value (normally values >3 are achieved) but high in comparison to the value obtained for tetanus toxoid (Pichler and Tilch, 2004). T-cell viability and reactivity were excellent as shown by pokeweed mitogen stimulation. This constellation reflects the immunosuppression by tacrolimus, which impairs T-cell receptor signaling, but still indicates T-cell stimulation by metamizole in this patient.

Pyrazolones such as metamizole are well-established allergens that can cause both immediate and delayed allergic responses (Ariza et al., 2016; Blanca-Lopez et al., 2016). Immediate allergic reactions include mainly urticaria, angioedema, and anaphylactic shock, whereas delayed reactions are mostly confined to the skin (Kowalski et al., 1998; Blanca-Lopez et al., 2016). Interestingly, immediate pyrazolone hypersensitivity has been associated with HLA-DQ and DR antigens (Kowalski et al., 1998), suggesting a genetic predisposition. Similarly, patients with metamizole-induced agranulocytosis had a higher frequency of the HLA-DQwl antigen as compared to patients with other types of agranulocytosis and healthy controls (Vlahov et al., 1996). In comparison, no association with HLA has so far been reported for pyrazolone-induced delayed skin reactions or hepatotoxicity. The fact that pyrazolone derivatives including metamizole are associated with delayed skin reactions supports the assumption that hepatotoxicity associated with metamizole may result from reactive T cells.

Based on recent studies suggesting a direct toxic effect of *N*-methyl-aminoantipyrine on granulocyte precursors (Rudin et al., 2019a; Rudin et al., 2019b), we also performed toxicological studies in two hepatocyte models. In HepaRG cells, we did not observe a relevant cytotoxicity, independently of CYP induction. Since we only checked the inducibility of the HepaRG cells but did not include a substance that becomes more toxic with CYP metabolism (Zahno et al., 2011), we cannot exclude with certainty the possibility of CYP-associated toxic metabolite formation *in vivo*. In HepG2 cells, we observed cytotoxicity in the presence of Fe^2+^ or hemin, but at MAA concentrations that are not reached *in vivo* and to an extent that appears not to be relevant. Furthermore, the findings regarding cellular ATP content and plasma membrane permeability were not consistent with each other, indicating that MAA and AA are not relevantly toxic for HepG2 and HepaRG cells. These findings are in agreement with those in mature granulocytes but are in contrast to the granulocyte precursor HL60 cells, which are sensitive to MMA in the presence of hemin (Rudin et al., 2019b). The reason for this discrepancy may be the antioxidative defense capacity, which is higher in hepatocytes and mature granulocytes than in granulocyte precursor cells (Rudin et al., 2019a). It is therefore possible that, depending on the cell type affected, metamizole can be cytotoxic by immunological or toxicological mechanisms.

## Data Availability

All datasets generated for this study are included in the manuscript.

## Author Contributors

All authors listed have made substantial, direct, and intellectual contribution to the work and approved it for publication.

## Funding

The work was supported by the endowment of the University of Basel and the University Hospital of Basel and by a grant of the Swiss National Science Foundation to SK (31003A_160206).

## Conflict of Interest Statement

WP was employed by ADR-AC GmbH.

The remaining authors declare that the research was conducted in the absence of any commercial or financial relationships that could be construed as a potential conflict of interest.
